# Phase 4 Pharmacovigilance Trial of Paromomycin Injection for the Treatment of Visceral Leishmaniasis in India

**DOI:** 10.1155/2011/645203

**Published:** 2011-11-17

**Authors:** Prabhat K. Sinha, T. K. Jha, Chandreshwar P. Thakur, Devendra Nath, Supriyo Mukherjee, Amrendra Kumar Aditya, Shyam Sundar

**Affiliations:** ^1^Rajendra Memorial Research Institute of Medical Sciences, Agam Kuan, Patna, Bihar 800 007, India; ^2^Kala-Azar Research Centre, Brahmpura, Muzaffarpur, Bihar, India; ^3^Balaji Utthan Sansthan, Patna, Bihar, India; ^4^Hazari Medical and Maternity Care, Motihari, Bihar, India; ^5^Research Centre for Diabetes, Hypertension, and Obesity, Samastipur, Bihar, India; ^6^Dr. A. K. Aditya Clinic, Samastipur, Bihar, India; ^7^Institute of Medical Sciences, Banaras Hindu University, Varanasi, Uttar Pradesh, India

## Abstract

*Background*. A phase 3 study demonstrated the safety and efficacy of paromomycin (paromomycin IM injection) for treatment of VL in an inpatient setting. *Methods*. This phase 4 study was conducted to assess the safety and efficacy of paromomycin in children and adults in an outpatient setting in Bihar, India. *Results*. This study enrolled 506 adult and pediatric patients. Of the 494 patients in the intent-to-treat (ITT) population, 98% received a full course of treatment. The overall study completion rate was 94% (462/494) for the ITT population and 96% (461/479) for the efficacy-evaluable (EE) population. Initial clinical cure was 99.6%, and final clinical cure 6 months after treatment was 94.2%. Grade 3 or 4 adverse events occurred in 5% of patients; events with a frequency of ≥1% were increases in alanine aminotransferase and aspartate aminotransferase. *Conclusions*. This study confirms the safety and efficacy of paromomycin to treat VL in an outpatient setting.

## 1. Introduction

Safe, effective, affordable, and accessible treatments are urgently needed for underserved populations in developing nations who are afflicted with one of the most neglected diseases: visceral leishmaniasis (VL), or kala-azar in South Asia. VL is endemic in 68 countries, primarily in the developing world, with an estimated at-risk population of 200 million [1]. As a systemic disease, VL causes chronic and irregular fever, weight loss, splenomegaly, hepatomegaly (less frequently), and anemia. If left untreated, VL is almost always fatal. New drugs are required for the treatment of VL partly because of the expansion of drug-resistant organisms [2–5].

Antimonials, which were historically the therapeutic mainstay for treating VL, have decreased effectiveness, especially in Bihar, India, where, in some cases, up to 65% of patients are estimated to fail antimonial treatment [3]. Pentamidine is toxic and has lost its initially high level of efficacy; it is no longer a recommended alternative treatment for VL [6]. The oral drug miltefosine is expensive and teratogenic, requiring the coadministration of oral contraceptives for 3 months in women of child-bearing age [7]. Although highly effective, amphotericin B is expensive, must be infused IV over 6 hours, is associated with fever, chills, and rigor, can be nephrotoxic, and requires close clinical and laboratory monitoring with hospitalization. Studies with lipid formulations of amphotericin B, such as AmBisome or Abelcet, have shown excellent results both in terms of safety and efficacy. A recent study showed that AmBisome (liposomal amphotericin B) is highly effective after a single IV infusion, and a new price agreement for developing countries has made AmBisome a more affordable treatment option [8]. However, because of a continuing concern regarding development of drug resistance, there continues to be a need for new, safe, efficacious, and low-cost therapies for the treatment of VL.

Paromomycin is an off-patent aminoglycoside antibiotic that has been marketed internationally as an oral, topical, and parenteral drug for bacterial and parasitic infections. In a phase 2 randomized, open-label, controlled trial of aminosidine (paromomycin) at different doses versus sodium stibogluconate for treating VL in North Bihar, India, paromomycin dosed at 16 mg/kg/day for 21 days cured 94% of cases at 6 months of followup [9, 10]. This study formed the basis for further development of this drug. Paromomycin was shown to be safe and effective for treatment of VL in a phase 3 clinical trial in India, with a final cure rate of 94.6% [11]. Paromomycin was subsequently registered for the treatment of VL in India in 2006 and was included in the WHO Essential Medicines List in 2007 [12, 13]. The safety profile of paromomycin along with the approved regimen to treat VL, intramuscular injection once daily for 21 consecutive days, should provide better access via the ability to treat patients in an outpatient setting. This purpose of this phase 4 study was to confirm the safety and efficacy profile observed in the phase 3 inpatient study in an outpatient setting.

## 2. Methods

A phase 4, open-label trial was conducted in two modules in the state of Bihar. Module 1 was a single-arm pharmacovigilance trial conducted at seven clinical sites in Bihar and enrolled 506 adult and pediatric VL patients aged 2–55 years (NCT00604955). The study was conducted by four research centers of excellence, including Rajendra Memorial Research Institute of Medical Sciences; the other three sites had prior experience conducting phase 4 studies. A 100-patient substudy using splenic and bone marrow aspirates for diagnosis and evaluation of cure was conducted at the Kalazar Research Centre, Muzaffarpur. The study was conducted in accordance with the current version of the Declaration of Helsinki, the Central Drugs Standard Control Organization's (CDSCO) Good Clinical Practices (GCP) For Clinical Research In India (2005), the Indian Council of Medical Research's (ICMR) Ethical Guidelines for Biomedical Research on Human Participants (2006), and Schedule Y of the Drug and Cosmetics Act and Rules (amended in 2005). Informed consent was obtained from all adult patients and the parent or legal guardian of children; when possible, children also provided assent. A consideration in conducting this clinical study was to expand capability to conduct clinical trials in rural India in accordance with the International Conference on Harmonisation (ICH) Guideline for GCP E6 (R1). An external advisory data and safety monitoring board (DSMB) was established to conduct periodic reviews of safety.

### 2.1. Patient Population

 Male and female patients aged 2 years (and weighing at least 5 kg) to 55 years with a clinical diagnosis of VL were eligible to participate in the study. Exclusion criteria included concurrent illness such as HIV, malaria, and tuberculosis; a history of hearing loss that could confound clinical detection of potential ototoxicity; recent or current exposure to medications that could result in compounded toxicities. Before study enrollment, informed consent was obtained from every patient or a legally authorized representative. After granting informed consent, patients with suspected VL had their diagnosis confirmed. For patients with no prior history of VL, a positive recombinant amastigote antigen K39 (rK39) test confirmed the diagnosis. The rK39 tests are easy to perform and cost effective, give reproducible results, and can therefore be used for early diagnosis of visceral leishmaniasis at both peripheral and central levels. It is specifically developed for field use and has good diagnostic accuracy in endemic areas [14]. For patients with a prior history of VL, a splenic or bone marrow aspirate with positive parasitology confirmed the diagnosis. In the 100-patient substudy, all patients had splenic or bone marrow aspirates to confirm diagnosis and parasitological cure.

### 2.2. Study Medication, Dose, and Mode of Administration

Paromomycin (paromomycin IM injection) was provided by the Institute for OneWorld Health, who sponsored the study. Paromomycin was manufactured by Gland Pharma Ltd., based in Hyderabad, India, and was formulated as a sterile, aqueous solution containing 375 mg/mL of paromomycin base in a single-use, 2 mL, amber, glass ampoule. 

Patients with a confirmed diagnosis of VL were screened for study eligibility within a 7-day screening period prior to initiation of drug therapy. Patients received the approved regimen for the treatment of VL in India: paromomycin, 11 mg/kg/day as the base, was administered by deep gluteal intramuscular injection once daily for 21 consecutive days (22 days if one injection was missed). The dosage (total volume in milliliters) of paromomycin was based on the patient's body weight in kilograms at the screening visit. Patients returned to the clinical site each day for treatment and safety assessments. As needed, to facilitate compliance and to relieve the financial burden of treatment, patients and one attendant each were provided financial support for transportation, food, and lodging.

If patients experienced treatment failure or relapse of VL, they were offered amphotericin B as an alternative (rescue) treatment for VL. Rescue treatment with amphotericin B required hospitalization for drug administration.

### 2.3. Safety and Efficacy Evaluations and Parameters

 During the 21 days of study drug treatment, safety assessments included daily assessment of treatment-emergent adverse events (AEs) and SAEs and injection site reactions. Additionally, patients underwent weekly evaluation of vital signs and laboratory assessments of renal and hepatic function. Although no formal audiology assessment was performed, patients were asked regularly about any subjective changes in hearing and any auditory and/or vestibular symptoms. Upon completion of the course of treatment (end of treatment [EOT]), all patients returned for safety assessments (AEs, laboratory evaluations, and vital signs) and efficacy assessment of initial clinical cure. Six months after EOT, patients returned to the clinical site for safety assessments (possibly related and related AEs) and efficacy assessment of final clinical cure. At any time during the initial 6 months after EOT, patients experiencing VL symptoms could return to the clinical site for an unscheduled visit. At unscheduled visits and at the scheduled visit 6 months after EOT, patients who exhibited clinical signs and symptoms of VL were identified and underwent a splenic or bone marrow biopsy to confirm disease relapse. Final clinical cure was defined as an initial clinical cure followed by the absence of new clinical signs and symptoms of VL through 6 months after EOT.

### 2.4. Statistical Analyses

Although a secondary objective of the study was to evaluate initial and final clinical cure, the study was not designed or statistically powered to definitively measure efficacy in this population. Because this was an open-label, single-arm study, no formal statistical hypothesis tests were planned. Descriptive statistics used to summarize the results were performed using SAS (version 9.1 or higher; SAS Institute) on a PC platform.

Safety analyses were based on the protocol-specified definition of the intent-to-treat (ITT) population, which included all patients who enrolled in the study and received at least one dose of paromomycin. Efficacy analyses were based on the efficacy-evaluable (EE) population, which included all patients without major protocol violations who received at least 20 doses of paromomycin.

## 3. Results

### 3.1. Patient Disposition and Demographics

**Figure 1:**
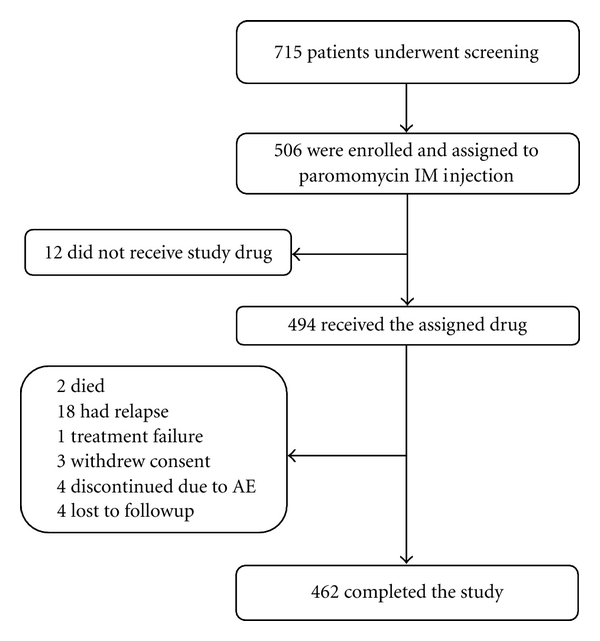
Disposition of patients in the intent-to-treat population.

 Of 715 screened patients, 506 met study entry criteria and were enrolled (Figure 1). Twelve enrolled patients did not receive study drug; data for these patients are not presented in this paper (five did not return to the clinic after screening, one withdrew consent, two were found to not meet the entry criteria upon return to the clinic, and four did not receive drug because the study had fully enrolled). The ITT population was 62% male, and 38% of patients were pediatric (2 to <15 years old) (Table 1). The overall study completion rate was 94% (462/494) for the ITT population and 96% (461/479) for the EE population. Treatment compliance was high; 485 (98%) patients received all 21 injections. An additional 15 patients were excluded from the EE population due to protocol violations, including patients who received fewer than 20 injections of study drug and patients for whom a 6-month final cure assessment was not obtained.

A total of 32 patients (6%) in the ITT population were prematurely discontinued from the study. The reasons for premature discontinuation were confirmed relapse (18/32; 56%), AE or nonfatal SAE (4/32; 13%), lost to followup (4/32; 13%), withdrawal of consent (3/32; 9%), death (2/32; 6%), and confirmed treatment failure (1/32; 3%). In the EE population, 18 patients (4%) discontinued study participation prematurely due to confirmed relapse (17/18; 94%) or treatment failure (1/18; 6%). Patient disposition, demographics, and disease history were similar across all clinical sites.

### 3.2. Efficacy

 Initial cure rate, which was assessed at EOT, was achieved in 99.6% (477/479; 95% CI, 98.5–99.9) patients in the EE population (Table 2). There was consistency across the clinical sites based on initial clinical cure rate, which ranged from 98.3% to 100.0%. Final clinical cure rate, which was assessed 6 months after EOT, was achieved in 94.2% (95% CI, 91.7–96.1) of 479 patients in the EE population (Table 2). Of note, data from this long-term clinical cure assessment was available for all but four patients in the ITT population who were lost to followup. All VL relapse (*N* = 18) and treatment failure (*N* = 1) patients were subjected to parasitological examination through demonstration of L.d. body in the splenic/bone marrow aspirates. After confirmation, the patients (*N* = 19) were administered with Amphotericin B in the dose of 1 mg per Kg body weight on alternate day for 15 infusions as per the study protocol. In subgroup analyses of efficacy based on age, sex, and clinical site, initial and final clinical cure rates across the subgroups were similar in both the EE and ITT populations, and final clinical cure rates were lower than initial clinical cure rates.

### 3.3. Safety and Tolerability

 Paromomycin was generally well tolerated when administered in an outpatient setting. In the ITT population, 77% of patients reported at least one treatment-emergent AE (Tables 3 and 4). Most patients (91%) who experienced an AE had an event with a maximum intensity of mild or moderate (i.e., CTCAE grade 1 or 2). The most frequently reported treatment-emergent AEs (i.e., incidence ≥10%) were increases in CTCAE grade of alanine aminotransferase (ALT) (52%), aspartate aminotransferase (AST) (46%), and alkaline phosphatase (16%) as well as injection site pain (10%). Transient increase in levels of hepatic enzymes, in Kala-Azar patients treated with aminoglycoside and paromomycin, has been reported in phase III clinical trial. This may be due to faster destruction of the parasites in liver tissue in patients [11]. Age and sex appeared to have no effect on the incidence of AEs, with the exception of injection site pain and increases in CTCAE grade of creatinine level, which were reported in a higher percentage of adults (defined as ≥15 to 55 years of age) than children (no formal statistical comparisons were made between subgroups).

Severe AEs (i.e., grade 3) included elevations in AST (18/494; 3.6%) and ALT (8/494; 1.6%), relapse of VL requiring hospitalization for the administration of amphotericin B (7/494; 1.4%), and single events of increased creatinine, increased bilirubin, injection site pain, injection site reaction, pneumonia, acute renal failure, tetany, anaemia, and anxiety. There were four life-threatening (i.e., CTCAE grade 4) AEs reported, three of which (sudden death, back pain with an outcome of death, and vaginal haemorrhage) the investigator considered unrelated to study drug. One patient had a CTCAE grade 4 (i.e., life-threatening) AE of AST increased (626.5 U/L; ALT, alkaline phosphatase, and total bilirubin were not determined) 45 days after EOT that the investigator classified as possibly related to study drug.

A total of 15 treatment-emergent SAEs were reported: two fatal events (0.4%) and 13 nonfatal events (2.6%). Neither of the two deaths was considered to be related to study drug. Two of the 13 nonfatal SAEs were considered related to study drug: acute renal failure and injection site reaction. Seven SAEs were relapsing VL that led to per-protocol hospitalization of patients for treatment.

Five (1%) patients treated with paromomycin were prematurely discontinued from study drug due to a treatment-emergent AE, including two events that were considered to be related to study drug (allergic dermatitis and increased total bilirubin level).

Other than the one SAE report of acute renal failure, renal function assessments showed no trend for deterioration. A mean decrease from baseline to each evaluation timepoint was observed for tests of renal function (i.e., blood urea nitrogen [BUN] and creatinine) and total bilirubin (Table 5). Mean increases from baseline were observed at all evaluation timepoints for transaminase and alkaline phosphatase levels. This is reflected in the proportion of patients with associated AEs that were reported in this study (52%, 46%, and 16% of patients with increased ALT, AST, and alkaline phosphatase, resp.). As was seen in the phase 3 trial [11], liver function test values were increased approximately 20% at weeks 1 and 2 but decreased toward baseline values at EOT. The majority of subjects had no deterioration from baseline in hepatic function indices. At the final evaluation, 79% of AST, 69% of ALT, 92% of alkaline phosphatase, and 99% of bilirubin values were either low/normal, or, if high, were high at both baseline and final evaluations. The proportion of subjects with shifts in transaminase levels from low/normal at baseline to high at the final evaluation was consistent across timepoints (18–21% for AST and 23–31% for ALT). The majority of subjects had either a decrease in toxicity grade or no change in grade; at the final evaluation, 73% of AST, 66% of ALT, 92% of alkaline phosphatase, and 99% of total bilirubin values were either the same or a grade decrease from the baseline value. Across all evaluations, only five (of 7,305; 0.07%) chemistry values increased more than two grades from the baseline value. Evaluation of vital signs throughout the study did not reveal concern associated with the administration of paromomycin. There were no reports of clinically detected hearing loss or patient complaints of hearing loss or vestibular symptoms.

### 3.4. Occurrence and Outcome of Pregnancies

Pregnant women were allowed to enroll in the study, and birth control was not regulated. Three female patients were pregnant at screening, and one female patient became pregnant more than 1 month after completing treatment. Two of the pregnant patients had AEs of increased AST and increased ALT, both grade 1. The other two pregnant patients did not report any AE. In all four pregnancy cases, the offspring were born alive and were determined to be normal/healthy just after birth.

## 4. Discussion

Paromomycin administered at 11 mg/kg daily for 21 consecutive days was generally safe and well tolerated. The efficacy of paromomycin was confirmed in this study based on long-term clinical cure in a large and demographically diverse group of outpatients with either new-onset or relapsing VL. The study population encompassed children (≥2 years), adolescents, and adults (≤55), including a small number of pregnant women. Of note, this study confirmed the efficacy and safety of PMIM in children below the age of 5, an age group not previously included in PMIM clinical studies.

Evidence for efficacy in this study is based on the high and consistent efficacy rates across analyses; the initial clinical cure at the EOT was 99.6% in the EE population, and final clinical cure, which was assessed 6 months after EOT, was 94.2% in the EE population. The final clinical cure rate is consistent with the final clinical cure rate of 94.6% observed in the phase 3 inpatient clinical study of paromomycin [11].

Increases in levels of liver enzymes have been reported with medications used to treat VL [4, 7, 15–17]. In this and prior studies [11] with paromomycin, a peak in liver function test values occurred after initiation of treatment and then declined toward baseline by EOT. It is unclear whether the hepatic effects of VL treatments are a direct result of the drugs or of a drug-disease interaction.

Although experience with use of paromomycin in pregnant women is limited, a teratological study of other aminoglycoside antibiotic use during pregnancy detected no teratogenic risk [18], and nonclinical studies have shown that paromomycin is not teratogenic, mutagenic, or genotoxic (PMIM product insert). Paromomycin may be used with caution in women of childbearing potential [18] (PMIM product insert); pregnant women were allowed to enroll in the study, and birth control was not regulated. In all four pregnancy cases in this study, the offspring were born alive and were determined to be normal/healthy just after birth. 

The effect of paromomycin on auditory and vestibular (cochlear) function has been assessed as part of more general toxicology studies of paromomycin in animals [19, 20]. The results of these animal studies indicate that paromomycin is associated with ototoxicity at high dose levels or at more moderate dose levels that are administered over extended periods of time. Paromomycin appears to have an auditory toxicity profile similar to that of amikacin and kanamycin; its vestibular effects are minor, occurring at high doses and in only a few animal species. Ototoxicity was not formally studied in this phase 4 study. There were no reports of clinically detected hearing loss or patient complaints of hearing loss or vestibular symptoms in this study although transient reversible ototoxicity was observed in 2% of subjects the phase 3 trial with paromomycin [11].

Although intramuscular treatments can be challenging for widespread public health use, patient compliance at the participating clinics was high. Food, lodging, and transportation were provided for each patient and a caregiver, which likely contributed to the high compliance rate. Primary health-center personnel have experience with intramuscular administration of other treatments, and supervised dosing may help decrease the risk of development of parasitic resistance caused by noncompliance.

## 5. Conclusions

In conclusion, paromomycin was shown to be efficacious when used in an outpatient setting. With the exception of increases in liver function test parameters, which decreased toward baseline over time, paromomycin has a reasonable safety profile, including demonstrated safety and efficacy in pediatric patients and, with limited data thus far, in pregnant women. Coupled with its relatively low cost, paromomycin is an accessible therapy for the treatment of VL.

## Figures and Tables

**Table 1:** Distribution of patients by age and sex.

Baseline characteristic	Efficacy-evaluable population (*N* = 479)	Intent-to-treat population (*N* = 494)
Age category, *n* (%)		
Pediatric (2 to <15 years)	187 (39%)	190 (38%)
2 to <5 years	19 (4%)	19 (4%)
5 to <15 years	168 (35%)	171 (35%)
Adult (≥15 to 55 years)	292 (61%)	304 (62%)
Sex, *n* (%)		
Male	295 (62%)	304 (62%)
Female	184 (38%)	190 (38%)

**Table 2:** Initial and final clinical cure rates by analysis population.

Analysis population	Initial clinical cure rate % (95% CI)	Final clinical cure rate % (95% CI)
Efficacy-evaluable population (*N* = 479)	99.6% (98.5–99.9)	94.2% (91.7–96.1)
Intent-to-treat population (*N* = 494)	98.4% (96.8–99.3)	91.5% (88.7–93.8)

CI, confidence interval.

**Table 3:** Summary of treatment-emergent adverse events.

Type of adverse event	Intent-to-treat population (*N* = 494)
Treatment-emergent adverse events, *n* (%)	
Any adverse event	379 (77%)
Treatment related	320 (65%)
Severe or life threatening^a^	35 (7%)
Led to premature discontinuation of study drug	5 (1%)
Serious, excluding death	13 (3%)^b^
Death, *n* (%)	2 (<1%)^c^

^a^Grade 3 or 4 according to National Cancer Institute (NCI) Common Terminology Criteria for Adverse Events (CTCAE) definition.

^b^Two events (injection site reaction and renal failure acute) were considered to be related to study drug.

^c^Both deaths were considered not related to study drug.

**Table 4:** Treatment-related and treatment-emergent adverse events in ≥1% of patients.

System organ class	CTCAE grade^a^					Total
Preferred term	Grade 1	Grade 2	Grade 3	Grade 4		(*N* = 494)
Any adverse event, *n* (%)	182 (37%)	116 (23%)	21 (4%)	1 (<1%)		320 (65%)
Investigations, *n* (%)						
Alanine aminotransferase increased	158 (32%)	49 (10%)	7 (1%)	0 (0%)		214 (43%)
Aspartate aminotransferase increased	92 (19%)	75 (15%)	17 (3%)	1 (<1%)		185 (37%)
Blood alkaline phosphatase increased	48 (10%)	11 (2%)	0 (0%)	0 (0%)		59 (12%)
Blood creatinine increased	26 (5%)	5 (1%)	1 (<1%)	0 (0%)		32 (6%)
Blood bilirubin increased	20 (4%)	0 (0%)	1 (<1%)	0 (0%)		21 (4%)
General disorders and administration site conditions, *n* (%)						
Injection site pain	18 (4%)	31 (6%)	1 (<1%)	0 (0%)		50 (10%)

^a^National Cancer Institute (NCI) Common Terminology Criteria for Adverse Events (CTCAE) definition.

**Table 5:** Mean change from baseline to study day 8, study day 15, and EOT/ET for tests of liver and renal function.

Indice	Mean (SD) Visit Value	Mean (SD) Change from Baseline
AST (U/L)		
Baseline (*N* = 494)	52.89 (27.16)	
Day 8 (*N* = 489)	73.54 (72.01)	20.47 (67.13)
Day 15 (*N* = 486)	64.36 (48.60)	11.26 (49.75)
EOT/ET^a^ (*N* = 486)	58.37 (26.21)	5.28 (32.65)
ALT (U/L)		
Baseline (*N* = 494)	39.62 (27.25)	
Day 8 (*N* = 489)	58.22 (70.62)	18.50 (62.96)
Day 15 (*N* = 486)	58.17 (54.35)	18.39 (52.00)
EOT/ET^a^ (*N* = 486)	51.83 (30.18)	12.05 (32.64)
Alkaline phosphatase (U/L)		
Baseline (*N* = 494)	229.00 (127.84)	
Day 8 (*N* = 489)	261.70 (168.80)	31.92 (129.97)
Day 15 (*N* = 486)	261.60 (137.59)	32.12 (114.93)
EOT/ET^a^ (*N* = 486)	254.23 (119.51)	24.75 (116.80)
Bilirubin total (mg/dL)		
Baseline (*N* = 494)	0.60 (0.26)	
Day 8 (*N* = 489)	0.57 (0.37)	−0.03 (0.38)
Day 15 (*N* = 486)	0.56 (0.23)	−0.03 (0.29)
EOT/ET^a^ (*N* = 486)	0.57 (0.22)	−0.03 (0.29)
BUN (mg/dL)		
Baseline (*N* = 494)	12.58 (4.15)	
Day 8 (*N* = 489)	10.20 (3.32)	−2.33 (4.55)
Day 15 (*N* = 486)	11.07 (3.64)	−1.46 (4.66)
EOT/ET^a^ (*N* = 486)	11.54 (4.39)	−0.99 (5.35)
Serum creatinine (mg/dL)		
Baseline (*N* = 494)	0.83 (0.26)	
Day 8 (*N* = 489)	0.75 (0.25)	−0.08 (0.20)
Day 15 (*N* = 486)	0.75 (0.26)	−0.09 (0.23)
EOT/ET^a^ (*N* = 486)	0.75 (0.30)	−0.09 (0.28)

ALT, alanine aminotransferase; AST, aspartate aminotransferase.

^a^End of treatment or early termination.

## References

[1] Narain JP, Dash AP, Parnell B (2010). Elimination of neglected tropical diseases in the South-East Asia Region of the World Health Organization. *Bulletin of the World Health Organization*.

[2] Lira R, Sundar S, Makharia A (1999). Evidence that the high incidence of treatment failures in Indian kala-azar is due to the emergence of antimony-resistant strains of *Leishmania donovani*. *Journal of Infectious Diseases*.

[3] Sundar S, More DK, Singh MK (2000). Failure of pentavalent antimony in visceral leishmaniasis in India: report from the center of the Indian epidemic. *Clinical Infectious Diseases*.

[4] Thakur CP, Narayan S, Ranjan A (2004). Epidemiological, clinical & pharmacological study of antimony-resistant visceral leishmaniasis in Bihar, India. *Indian Journal of Medical Research*.

[5] Das VNR, Ranjan A, Bimal S (2005). Magnitude of unresponsiveness to sodium stibogluconate in the treatment of visceral leishmaniasis in Bihar. *National Medical Journal of India*.

[6] World Health Organization (2010). *WHO Model List of Essential Medicines*.

[7] Sundar S, Jha TK, Thakur CP (2002). Oral miltefosine for Indian visceral leishmaniasis. *The New England Journal of Medicine*.

[8] Sundar S, Chakravarty J, Agarwal D, Rai M, Murray HW (2010). Single-dose liposomal amphotericin B for visceral leishmaniasis in India. *The New England Journal of Medicine*.

[9] Jha TK, Olliaro P, Thakur CPN (1998). Randomised controlled trial of aminosidine (paromomycin) v sodium stibogluconate for treating visceral leishmaniasis in North Bihar, India. *British Medical Journal*.

[10] Thakur CP, Kanyok TP, Pandey AK (2000). A prospective randomized, comparative, open-label trial of the safety and efficacy of paromomycin (aminosidine) plus sodium stibogluconate versus sodium stibogluconate alone for the treatment of visceral leishmaniasis. *Transactions of the Royal Society of Tropical Medicine and Hygiene*.

[11] Sundar S, Jha TK, Thakur CP, Sinha PK, Bhattacharya SK (2007). Injectable paromomycin for visceral leishmaniasis in India. *The New England Journal of Medicine*.

[12] World Health Organization (2007). *WHO Model List of Essential Medicines*.

[13] World Health Organization (2007). *WHO Model List of Essential Medicines for Children*.

[14] WHO (2010). Control of the leishmaniasis: report of a meeting of the WHO expert Committee on the Control of Leishmaniasis.

[15] Jha TK, Sundar S, Thakur CP (1999). Miltefosine, an oral agent, for the treatment of indian visceral leishmaniasis. *The New England Journal of Medicine*.

[16] Sundar S, Rai M, Chakravarty J (2008). New treatment approach in Indian visceral leishmaniasis: single-dose liposomal amphotericin b followed by short-course oral miltefosine. *Clinical Infectious Diseases*.

[17] Bhattacharya SK, Sinha PK, Sundar S (2007). Phase 4 trial of miltefosine for the treatment of Indian visceral leishmaniasis. *Journal of Infectious Diseases*.

[18] Czeizel AE, Rockenbauer M, Olsen J, Sorensen HT (2000). A teratological study of aminoglycoside antibiotic treatment during pregnancy. *Scandinavian Journal of Infectious Diseases*.

[19] Arcamone F, Bertazzoli C, Buogo A

[20] Di Marco A, Bertazzoli C Pharmacology of new basic oligosaccharide antibiotics.

